# Use of Primary Care during the Year before Childhood Cancer Diagnosis: A Nationwide Population-Based Matched Comparative Study

**DOI:** 10.1371/journal.pone.0059098

**Published:** 2013-03-12

**Authors:** Jette Møller Ahrensberg, Morten Fenger-Grøn, Peter Vedsted

**Affiliations:** 1 The Research Unit for General Practice, Aarhus University, Aarhus, Denmark; 2 Section for General Practice, Department of Public Health, Aarhus University, Aarhus, Denmark; 3 Research Centre for Cancer Diagnosis in Primary Care, Aarhus University, Aarhus, Denmark; Dartmouth, United States of America

## Abstract

**Objective:**

Childhood cancer is rare and symptoms tend to be unspecific and vague. Using the utilization of health care services as a proxy for symptoms, the present study seeks to determine when early symptoms of childhood cancer are seen in general practice.

**Methods:**

A population-based matched comparative study was conducted using nationwide registry data. As cases, all children in Denmark below 16 years of age (N = 1,278) diagnosed with cancer (Jan 2002-Dec 2008) were included. As controls, 10 children per case matched on gender and date of birth (N = 12,780) were randomly selected. The utilization of primary health care services (daytime contacts, out-of-hours contacts and diagnostic procedures) during the year preceding diagnosis/index date was measured for cases and controls.

**Results:**

During the six months before diagnosis, children with cancer used primary care more than the control cohort. This excess use grew consistently and steadily towards the time of diagnosis with an IRR = 3.19 (95%CI: 2.99–3.39) (p<0.0001) during the last three months before diagnosis. Children with *Central Nervous System* (CNS) tumours had more contacts than other children during the entire study period. The use of practice-based diagnostic tests and the number of out-of-hours contacts began to increase four to five months before cancer diagnosis.

**Conclusions:**

The study shows that excess health care use, a proxy for symptoms of childhood cancer, occurs months before the diagnosis is established. Children with lymphoma, bone tumour or other solid tumours had higher consultation rates than the controls in the last *five* months before diagnosis, whereas children with CNS tumour had higher consultation rates in all *twelve* months before diagnosis. More knowledge about early symptoms and the diagnostic pathway for childhood cancer would be clinically relevant.

## Introduction

Childhood cancer is rare and affects 1 in 5–600 children in Western countries. However, although rare, it is, next to accidents, the second most common cause of childhood death in developed countries [1]. Children with early stage cancer often present with non-specific symptoms or symptoms that do not indicate serious disease [2]–[3], but mimic common conditions, such as infections, developmental processes or psychological problems [4]–[5]. In childhood cancer in particular, alarm symptoms seem to have very low positive predictive values [6], and the diagnostic process is challenging in general practice where the risk of serious disease in children is real, but very low [7]. It is therefore important to obtain knowledge about children’s health care seeking behaviour prior to a cancer diagnosis. Such knowledge may be obtained from general practitioners (GPs) who often report that the diagnostic intervals are short in childhood cancer [8].

In Denmark, the GP acts as a gatekeeper and provides frontline medical advice [9]. Thus, the GP is the first point of contact on the diagnostic pathway and much research has been conducted to explore the GP’s role in diagnosing cancer [9]–[12], but surprisingly little is known about the nature of children’s cancer pathway in general practice and the particular challenges posed by their presenting symptoms. A few studies suggest that these children tend to attend general practice more than children in the background population [3], [6], [13].

Studies of health care seeking behaviour prior to a childhood cancer diagnosis may contribute new knowledge about the ‘diagnostic time window’. In this first nationwide population-based matched comparative study using complete registry data, we aimed to explore patterns in primary care use during the year preceding a cancer diagnosis, accounting for timing, cancer type, age and gender.

## Methods

### Study Design and Study Population

We conducted a population-based matched comparative study using information from three nationwide registries: the *Danish Cancer Registry* (DCR) which holds information on all cancer diagnoses in Denmark [14]; the *Danish Civil Registration System* (CRS) which has updated information on all Danish citizens [15]; and the *Danish National Health Insurance Service Registry* (NHSR) which holds information on all contacts to general practice and all services provided [16]. The civil registration number (CRN), a unique 10-digit personal identification number assigned to every Danish citizen at birth, was used to link registers on the level of the individual. The registers are known to be very valid and complete [17].

**Figure 1:**
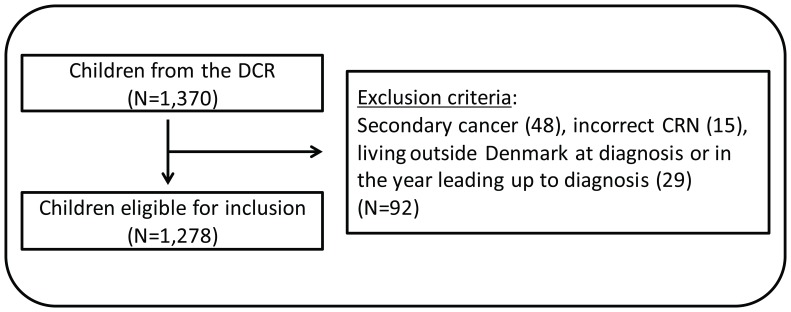
The sampling of childhood cancer patients.

All 0–15-year-old children with incident cancer according to the Danish version of the International Classification of Diseases ICD-10 (ICD-10) (Chapter II, C00-D48) [18] between 1 January 2002 and 31 December 2008 (N = 1,370) were identified in the DCR. A total of 92 children were excluded because of secondary cancer diagnosis (N = 48), incorrect CRN (N = 15), residence outside Denmark on the date of diagnosis or during the whole or part of the year leading up to their diagnosis (N = 29). Thus, 1,278 children were included as cases (Figure 1).

For comparison, ten randomly selected children were sampled per cancer case (N = 12,780) using the CRS. They were matched on gender and date of birth. These controls were alive, without a history of cancer on the day the case was diagnosed with cancer (incidence density sampling) [19] and resident in Denmark on the date of diagnosis/index day and throughout the year preceding the diagnosis. A control subject could only be sampled once per case, but could be both a control and potentially also a case later in the study period.

### Primary Health Care Services

The main outcome of the study was rates of consultations and diagnostic tests. Information on health care utilization during the 12 months prior to the diagnosis/index day was obtained from the NHSR in the same matter for both cases and controls. All primary health care services provided to citizens in Denmark are registered prospectively in the NHSR with specific codes, and the child’s CRN can be linked to the unique practice identification number assigned to the general practice with which the patient is listed. The registration is based on a fee-for-service remuneration to the provider and is thus very complete [16]. The NHSR provided data on consultations in daytime and out-of-hours (OOH) and on diagnostic tests performed during the daytime the year before the diagnosis/index day for all study participants. Daytime contacts included all main contacts (consultations, home visits, email and telephone consultations). The preventive consultations and vaccinations made by GPs in Denmark were not included.

Daytime diagnostic tests performed in practices included blood tests (CRP test, differential blood count, blood glucose test, haemoglobin and blood tests sent from the GP to a laboratory), urine tests (urine test by stick, microscopy of urine and urine culture), pulmonary function test, ECG and tests for streptococcal throat infection. The OOH contacts included consultations, telephone consultations and home visits. Telephone consultations followed by a consultation or a home visit were excluded.

Consultation rates were measured as mean consultations per month (graphic presentations) and as consultations per three months intervals (tables). The diagnostic tests were measured as rates, e.g. mean diagnostic tests/month (graphs) and mean diagnostic tests/three months (tables).

### Statistical Analyses

For children with cancer and for the control subjects, the monthly and quarterly rates and the rate ratios between the two groups’ daytime contacts, daytime diagnostic tests and OOH contacts during the study period were calculated. 95%-confidence intervals (95% CIs) were assessed using a negative binomial regression model applying cluster robust variance estimation to account for heterogeneity between subjects [20]. A two-group effect of gender and a linear effect of age (on the implied log-scale) were included in the models. According to the ICD10 codes, cases were divided into five subgroups (A–E): A: leukaemia (C91–C95), B: lymphoma (C81–85+ C96); C: CNS tumour (C70–72, C75.1–3, D32–33+ D35.2–4, D42–43, D44.3–5); D: bone tumour (C40–41), and E: other solid tumour (all remaining ICD10 codes, Chapter II). Subgroup analysis was made for each of the five cancer subgroups (A–E) and for their corresponding control subjects. Curves for the dates of the latest GP visits before diagnosis and associated confidence bands were drawn by applying a standard Kaplan-Maier procedure and normal approximation on a reversed time scale. The percentage of children with four or more daytime contacts during the three months prior diagnosis was calculated. Data were analysed using the statistical software Stata 12.0 (StataCorp LP, TX, USA).

### Ethics

The study was approved by the Danish Data Protection Agency (J.no. 2008-41-2956). According to the Central Denmark Region Committees on Biomedical Research Ethics, the Act on Research Ethics Review of Health Research Projects did not apply to this project.

## Results

### Characteristics of the Participants

The characteristics of the two matched groups (cases and controls) are shown in Table 1. At the time of data collection (median 1823 days; IQI 1278–2551 after diagnosis/index day), 189 (15.6%) children diagnosed with cancer had died. Of these deaths, 81 (42.9%) occurred within the first year following the diagnosis. In the control group, six children had died (0.05%).

**Table 1:** Descriptive data on the childhood cancer patients.

	Leukaemia		Lymphoma		CNS tumour		Bone tumour		Other solid tumour		Total	
	N	(%)	N	(%)	N	(%)	N	(%)	N	(%)	N	(%)
**All**	354	(27.7)	105	(8.2)	298	(23.3)	65	(5.1)	456	(35.7)	1278	(100.0)
**Gender**												
Boys	191	(28.6)	64	(9.6)	148	(22.2)	28	(4.2)	236	(35.4)	667	(100.0)
Girls	163	(26.7)	41	(6.7)	150	(24.5)	37	(6.1)	220	(36.0)	611	(100.0)
**Age at diagnosis**												
0 years	23	(20.2)	0	(0.0)	18	(15.8)	0	(0.0)	73	(64.0)	114	(100.0)
1–4 years	178	(44.2)	10	(2.5)	78	(19.4)	3	(0.7)	134	(33.3)	403	(100.0)
5–9 years	85	(28.4)	30	(10.0)	90	(30.1)	19	(6.4)	75	(25.1)	299	(100.0)
10–15 years	68	(14.7)	65	(14.1)	112	(24.2)	43	(9.3)	174	(37.7)	462	(100.0)

For each childhood cancer patient, ten control persons were included matched on age and gender.

### General Practice Consultations during Daytime

**Figure 2:**
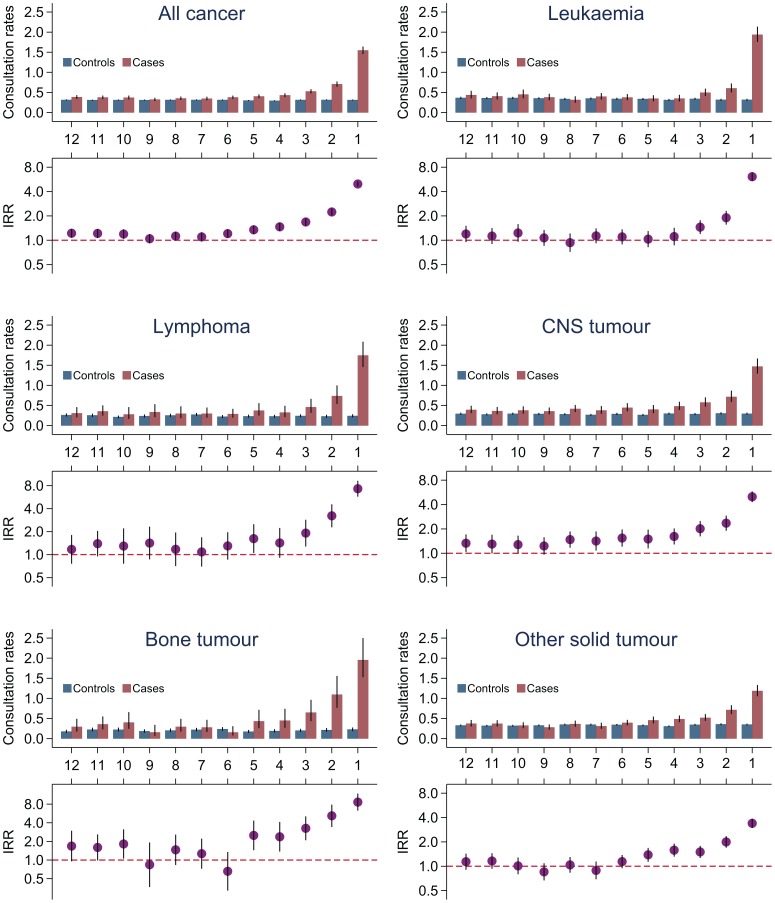
Daytime consultations in general practice. Upper part: Consultation rates (mean consultations per month) in general practice for children with cancer and control children the year before diagnosis/index day. Lower part: The incidence rate ratios (IRR) for consultations with 95% confidence intervals (95%CIs).

During the year before diagnosis, childhood cancer patients had a higher monthly rate of daytime consultations in primary care than controls (Figure 2 and Table 2). A statistically significant and progressive increase in childhood cancer patients’ daytime consultation rates was observed the last six months before the diagnosis, especially during the last three months (IRR = 3.19, 95%CI: 2.99–3.39) (p<0.001). The absolute *excess* number of daytime consultations in the last three months before diagnosis was two, but children with bone tumours had an excess of three consultations. Remarkably, the curve seemed to be slightly bimodal: cases’ utilization was statistically significantly higher than the controls’ at the start of the study period, then their utilization equalled the controls’ and, finally, their utilization rose again.

**Table 2:** Rates for consultations and diagnostic tests in daytime for children with cancer and control persons.

	Consultations, daytime			Diagnostic tests		
	Cases	Controls	IRR	Cases	Controls	IRR
**All Cancers**						
10–12 months	1.17	0.96	1.22 (1.11–1.34)††	0.16	0.12	1.33 (1.07–1.65)*
7–9 months	1.06	0.97	1.10 (1.01–1.20)*	0.13	0.12	1.03 (0.80–1.34)
4–6 months	1.22	0.92	1.37 (1.26–1.49)††	0.14	0.12	1.24 (0.96–1.60)
1–3 months	2.79	0.95	3.19 (2.99–3.39)††	0.74	0.13	5.62 (4.83–6.53)††
**Leukaemia**						
10–12 months	1.31	1.10	1.19 (1.01–1,41)*	0.17	0.11	1.56 (1.07–2.26)*
7–9 months	1.13	1.08	1.05 (0.90–1.22)	0.12	0.11	1.05 (0.71–1.56)
4–6 months	1.08	1.00	1.07 (0.92–1.25)	0.10	0.11	0.86 (0.56–1.32)
1–3 months	3.04	0.98	3.34 (3.00–3.71) ††	1.00	0.12	8.01 (6.37–10.08)††
**Lymphoma**						
10–12 months	0.93	0.73	1.28 (0.94–1.75)	0.09	0.11	0.72 (0.28–1.86)
7–9 months	0.92	0.76	1.21 (0.89–1.64)	0.09	0.12	0.75 (0.27–2.12)
4–6 months	0.98	0.68	1.48 (1.11–1.99)*	0.14	0.13	1.08 (0.52–2.26)
1–3 months	2.93	0.71	4.27 (3.57–5.10)††	1.21	0.11	10.98 (7.01–17.21)††
**CNS tumour**						
10–12 months	1.15	0.87	1.32 (1.11–1.57)*	0.18	0.13	1.40 (0.92–2.13)
7–9 months	1.20	0.87	1.42 (1.19–1.70)†	0.16	0.13	1.31 (0.73–2.34)
4–6 months	1.33	0.86	1.60 (1.35–1.88)††	0.23	0.11	2.00 (1.22–3.28)*
1–3 months	2.78	0.89	3.35 (2.94–3.82)††	0.53	0.14	4.00 (2.80–5.70)††
**Bone tumour**						
10–12 months	1.05	0.62	1.69 (1.18–2.42)*	0.20	0.12	1.75 (0.81–3.79)
7–9 months	0.72	0.60	1.19 (0.81–1.75)	0.12	0.09	1.28 (0.54–3.02)
4–6 months	1.03	0.59	1.72 (1.19–2.51)*	0.05	0.10	0.48 (0.15–1.53)
1–3 months	3.69	0.64	5.85 (4.53–7.55)††	0.77	0.14	5.87 (2.87–11.98)††
**Other solid tumour**						
10–12 months	1.14	1.02	1.11 (0.94–1.32)	0.14	0.13	1.20 (0.78–1.84)
7–9 months	1.00	1.07	0.92 (0.78–1.07)	0.12	0.14	0.89 (0.59–1.34)
4–6 months	1.36	1.00	1.40 (1.22–1.61)††	0.14	0.12	1.14 (0.76–1.70)
1–3 months	2.44	1.06	2.47 (2.22–2.76)††	0.56	0.14	3.90 (2.95–5.15)††

Rates (mean number of consultations/diagnostic tests per 3 months interval) and incidence rate ratios (IRR) for children with cancer and control persons the year before diagnosis (for cases)/index day (for controls). The IRRs are presented with 95% confidence intervals (95%CIs).

* p<0.05.

† p<0.001.

†† p<0.0001.

**Figure 3:**
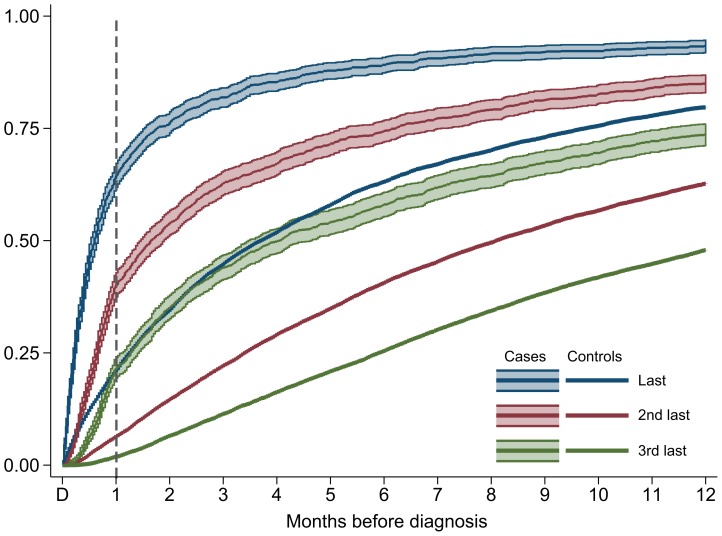
Childhood cancer patients and controls in general practice during the 12 months before diagnosis. The curves show the proportion with the latest, the second latest and third latest consultation (y-axis) the year before cancer diagnosis (x-axis). For comparison, the same is shown for the matched control children until the index day. E.g. 64% of the children with cancer are seen once within a month before the diagnosis, whereas this figure is 21% for the control children.

A total of 93.3% of the childhood cancer patients and 79.7% of the controls had consulted the GP within the preceding year, and 81.8% of the former and 44.8% of the latter had consulted the GP within three months before diagnosis (Figure 3). Looking backward from the date of diagnosis, the GPs had been consulted twice by half of the cancer patients 45 days before diagnosis, and 50% of the children with cancer had consulted three times within four months. Half of the controls had been seen twice within eight months before the index day and three times within more than a year.

Among children with cancer, those who had lymphoma, bone tumours and other solid tumours had higher consultation rates than the controls the last *five* months before diagnosis (Figure 2), whereas children with CNS tumours had higher consultation rates all *twelve* months before diagnosis. Leukaemia patients had higher consultation rates the last *three* months before diagnosis (IRR = 3.34, 95%CI: 3.00–3.71) (p<0.001) (Table 2). It should be noted that a statistically significant increase in the consultation rate nine to twelve months before diagnosis (bimodal curve) was also observed among children with leukaemia or bone tumours (Figure 2).

The percentage of children who consulted the GP four times or more during the three month before diagnosis/index day was 30.6% among cases vs. 6.1% among controls (likelihood ratio = 5.1, 95%CI: 4.5–5.6). The highest likelihood of ≥4 consultations was observed among children subsequently diagnosed with a bone tumour (Table 3).

**Table 3:** Percentages of children with four or more consultations in general practice during the three months before diagnosis and likelihood ratio of four or more consultations.

	Percentage of childrenwith ≥4 consultations		LikelihoodRatio (95% CI*)
	Cases (%)	Controls (%)	
All Cancers	30.6	6.1	5.1 (4.5–5.6)
Leukaemia	36.4	6.3	5.8 (4.8–7.0)
Lymphoma	33.3	3.7	9.0 (6.0–13.5)
CNS tumour	29.9	5.6	5.4 (4.3–6.8)
Bone tumour	43.1	3.1	14.0 (8.4–23.4)
Other solid tumour	24.1	7.2	3.4 (2.8–4.1)

* CI = confidence interval.

### Diagnostic Procedures in Daytime and Out-of-hours Consultations

**Figure 4:**
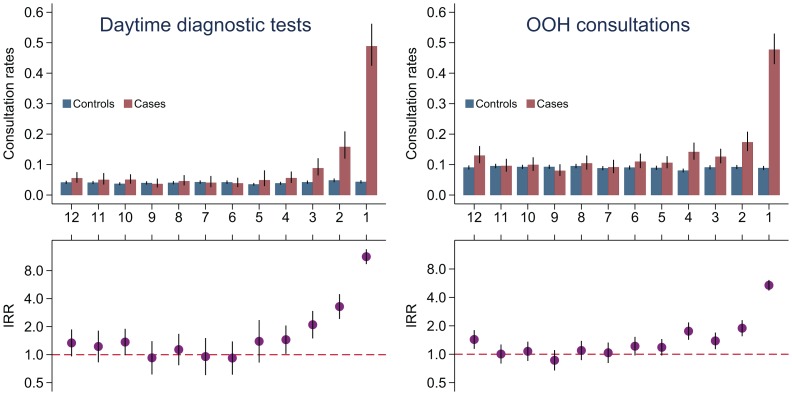
Daytime diagnostic tests and out-of-hours consultations. Upper part: Rates (mean tests per month) for diagnostic tests and OOH consultations (mean per month) for children with cancer and control children the year before diagnosis/index day. Lower part: Incidence rate ratios (IRR) for diagnostic tests and OOH consultations with 95% confidence intervals (95%CIs).

Overall, the rates for diagnostic procedures were statistically significantly higher for children with cancer than for controls in the three months before diagnosis (IRR = 5.62, 95%CI: 4.83–6.53) (p<0.001) and statistically higher during the last six months before the diagnosis of a CNS tumour (Table 2 and Figure 4). A small increase in the utilization of diagnostic procedures was also observed nine to twelve months before diagnosis. During the four months before diagnosis, a statistically significant increase in use of OOH contact rates among childhood cancer patients was observed (Figure 4).

## Discussion

### Main Findings

More than 80% of children consulted the GPs within three month before receiving a cancer diagnosis and 95% within a year. During the last six months before diagnosis, their excess consultation rate rose consistently and steadily with decreasing time to diagnosis. During the last three months before diagnosis, the children had a three-fold higher use of primary health care than their controls. This was consistent across all cancer types. The excess of daytime consultations was highest (three) for children with bone tumours; and the average excess of consultations was two the last quarter before diagnosis. However, children with brain tumours consulted the GP more than controls during the entire year until diagnosis, whereas the consultations rates for children with bone tumours, lymphoma and other solid tumours were similar to those of the control children up until five months before the diagnosis. For children with leukaemia, the use of general practice began to increase three month before diagnosis. Childhood cancer patients also had a marked use of OOH services during the last five months before diagnosis. The utilization of diagnostic procedures began to rise three months prior to diagnosis. During the last three months before diagnosis, 30.6% of children with cancer, and 43.1% of children with a bone tumour had four or more consultations vs. 5.1% and 3.1% of controls, respectively.

### Strengths and Weaknesses

Our population-based study obtained complete information on prospectively recorded primary health care use for all children diagnosed with cancer in Denmark during an eight-year period and for a random sample of control children of the same age and gender. The data were thus not collected for the purpose of the present study and were independent of the memory of GPs or study participants. All of the consultations and diagnostic procedures measured in the study were provided or requested by GPs and available to the patients free of charge. Consultations and diagnostic procedures are coded for remuneration because about 75% of the GP’s salary is based on fee-for-services. The accuracy and the completeness of data in the NHSR is therefore high [16]. Our information on childhood cancer in the DCR was registered prospectively, and the cancer diagnosis was based on the WHO classification and coded by the physician in charge of the discharge. The DCR has been shown to be accurate and to have a nearly complete registration of cancer cases [21]. As from 1 January 2004, the exact date of diagnosis has been recorded in the DCR based on the international hierarchy that uses the dates of histological confirmation, admission to hospital and date of death. Until 2004, the first day in the month of hospital admission was used as the date of diagnosis if no histological diagnosis was available. The primary health service utilization during the month before the diagnosis may therefore have been underestimated for children diagnosed in 2002 and 2003 and for their respective control persons. These potential misclassifications were equally distributed among cases and control persons (same index day as the date of diagnosis for cases) and the effect, if any, is therefore likely to be small. Selection bias and information bias in relation to diagnosis and health care services are hence negligible in the present study.

Although childhood cancers are rare, we obtained a relatively high statistical precision which made it possible to detect small, but clinically relevant differences between the groups.

We eliminated the effect of confounding by age and gender by matching cases with controls. However, we cannot exclude that residual confounding by other factors may play a role. We had no information on pre-existing co-morbidity, but co-morbidity is relatively rare among children, and is expected to play no significant role in this study. Few genetic disorder and syndromes [22]–[23] can predispose to malignancies in childhood and in such cases, the child may already be a frequent attender in general practice. This would tend to overestimate the association between the consultation frequency and early stage cancer. These conditions are very rare and their influence is minimal, if any, on the present study.

No information on the reason for the encounter or the signs found by the GPs was available. Our results thus call for future studies to describe early cancer symptomatology and the reasons for e.g. making diagnostic tests on children months before their diagnosis is established. The nationwide approach allows us to consider the results as generalizable and applicable in countries where patients enter the healthcare system via primary care.

### Comparison with Other Studies

A recent study [6] showed an increase in consultation rates among childhood cancer patients in the year before diagnosis, and most of this increase was seen in the three months preceding diagnosis. In the same 3-months time window, 35.5% of cases had four or more consultations to general practice compared with 9.1% among controls. This is in keeping with our findings for the total group of children with cancer. The study also demonstrated very low predictive values for symptoms of childhood cancer [6]. A previous Swedish study illustrated a higher consultation rate in general practice the year before diagnosis in childhood cancer patients (2.3 times for leukaemia, 1.5 times for CNS tumour) than in age- and sex-matched controls [3], and it indicated that symptoms may appear long before diagnosis is made. Furthermore, a British study reported a higher frequency of disease-relevant symptoms and a higher consultation rate several years before the diagnosis for children with brain tumours than for controls [13]. A study on 74 children with brain tumours showed an average of 4.6 (range 1–12) consultations with professionals before diagnosis was made [24]. Also, previous studies have showed a longer time interval from symptom onset to diagnosis for children diagnosed with tumours in the CNS [24]–[27] and bone [5], [8], [28]–[29] than for children diagnosed with leukaemia. The present study stresses the importance of recognition of symptoms or signs that make children attend more frequently. It is demonstrated that the GPs acted upon symptoms by using practice-based diagnostic tests. This indicates that a closer examination of these tests and test usage patterns may help identify those who should be suspected of having cancer. The present study also makes clear that the very short time interval from the first presentation to diagnosis reported by GPs [8] may not reflect optimal clinical knowledge about symptom presentation.

### Conclusion and Implications

In this nationwide study including 0–15-year-old children with cancer, a markedly increased use of general practice in daytime and OOH appeared in the months before diagnosis. Such excess use of primary health care services was considered a proxy for symptom presentation. Increased use of health care services close to diagnosis is largely what should be expected as it mirrors an increased diagnostic activity level. However, already six months before diagnosis, children with cancer began to make progressively more use of primary health care. Children with CNS tumours used general practice more than controls throughout the entire year leading up to the diagnosis. The present study indicates that the symptoms of some childhood cancers seen in primary care do not seem to invite suspicion, and the absence of such early suspicion is one of the factors that prolong the diagnostic pathway.

The study found a minor excess of consultations for children with cancer already 10–12 months prior to diagnosis. Further studies are needed to explore possible reasons for this *bimodal* pattern of consultation rates.

The study also shows an excess of consultations during the last quarter before diagnosis where the GP might, and perhaps did, suspect a serious disease as evidenced by the tendency to make more diagnostic tests several months before the diagnosis. Parents may continue to return to the GP although the GP is unable to identify a specific problem [4], and the present study emphasizes the need for obtaining a detailed medical history in children presenting with vague or persistent symptoms. Such a history could help ensure timely diagnosis of this very rare condition in general practice. Future research should aim to investigate how presenting symptoms and symptom interpretation influence the time interval from the first symptom presentation to diagnosis.
